# Effects of varying concentrations of Ubiquinol (coenzyme Q10) on physiological parameters in male donkeys (*Equus asinus*)

**DOI:** 10.3389/fvets.2026.1733317

**Published:** 2026-02-16

**Authors:** Muhammad Faheem Akhtar, Muhammad Umar, Wang Changfa

**Affiliations:** 1 Liaocheng Research Institute of Donkey High-Efficiency Breeding and Ecological Feeding, Liaocheng University, Liaocheng, China; 2 Department of Animal Reproduction, Faculty of Veterinary and Animal Sciences, Lasbela University of Agriculture, Water and Marine Science, Uthal, Pakistan

**Keywords:** China, male donkeys, non-seasonal breeeding, Q10, Ubiquinol feeding

## Abstract

Coenzyme Q10 (CoQ10) serves as an antioxidant, and it is considered an energy source. Ubiquinol inhibits lipid peroxidation and protein oxidation. We fed CoQ10-ubiquinol in donkeys’ feed and investigated its effect on plasma hormone concentrations of Testosterone (T), Activin-A, Luteinizing Hormone (LH), follicle-stimulating hormone (FSH), Antimullerian hormone (AMH), estradiol (E2), and Q10 in donkeys. For this purpose, Dezhou jacks, *n* = 30, were equally divided into groups A, B, and C. Group A donkeys were given 2 g Uboquinol (CoQ10)/day. Group B received 1.5 g CoQ10/day, and group C served as the control group. Experimental duration was 56 days. The objective of the present study was to check the effect of various concentrations of Coenzyme Q10 (Ubiquinol) on plasma hormones that ultimately affect semen quality in donkeys, especially during the non-breeding season. Results indicate that the addition of Ubiquinol in donkey feed has a tendency to improve plasma hormone concentrations of T, FSH, LH, AMH, Activin-A, E2, and Q10. Our findings suggested that feeding 2 g of Ubiquinol/day mixed into 1 pound of grain-based concentrate with molasses improved plasma hormone concentrations. In conclusion, our findings suggest that feeding CoQ10 may improve donkey semen quality.

## 1 Introduction

Reproductive quiescence is a salient feature of seasonal breeder animals. The effect of seasonality on reproductive efficiency is highly controversial (1). Donkeys are long-day breeders. Short and long days highly influence reproductive efficiency, including semen quality. However, reproductive efficiency in seasonal breeders is also linked to nutrition, breed, environment, and health (2). In commercial donkey farming in China, female donkeys already have low conception rates as compared to other farm animal species, including cattle, goats, sheep, etc. Coupled with low reproduction in the low breeding season (November–February), results in economic loss in terms of low foal production per year. Semen quality determines conception rates in mares (3). Semen quality declines in non-seasonal breeders (4). Similarly, heat stress also lowers semen quality during the summer months. In our recent work (5), we examined the effects of temporary heat stress in donkeys. Short-term heat stress lowered plasma hormone concentrations of Testosterone (T) and Luteinizing Hormone (LH) and reduced germ cells.

The human population is expected to reach 9.3 billion by 2050. Such a rapid increase in population will also result in higher food requirements. High-quality animal farm products cannot be achieved without focusing on fodder quality, feed quality, animal husbandry practices, and reproduction (6). Among these animal products, donkey meat and milk have emerged as significant nutritional resources, particularly in China. Donkey meat and milk are quite popular in China and other parts of the world. However, its population in China has significantly declined in recent years. Given the industry’s declining trend, the Government of China has prioritized donkey conservation, and owing to donkey meat and milk’s nutritional benefits, its population is now improving (1). Conception rates in mares are highly affected by semen quality in stallions (7, 8). Age, animal husbandry practices, disease management, herd health affect semen quality, and seasonal factors. Low semen quality, coupled with the breeding season, reduces economic gains in the donkey industry. Donkey fertility drops significantly outside of the breeding season. In the past, dietary supplementation with antioxidants, alone or in combination with polyunsaturated fatty acids, has improved semen quality in various species, e.g., humans, rats, poultry, swine, and equids (9–14). Coenzyme Q10 is a lipid-soluble benzoquinone compound that acts as an electron carrier in the mitochondrial respiratory chain (15). CoQ10 is a naturally occurring compound that has antioxidant properties. Coenzyme Q10 proved effective against testicular injury in mice exposed to a magnetic field (16, 17). Concentrated CoQ10 powder improved the semen quality of infertile men (18–21). Post-thaw semen quality in stallion semen was improved after CoQ10 addition (22). Coenzyme has anti-inflammatory and antioxidative properties (23, 24). Extensive research has documented various positive effects of the CoQ10 enzyme, but its specific effect on donkey reproduction has not been elaborated upon so far. Therefore, our study aims to determine the effects of feeding CoQ10 enzyme on plasma hormone concentrations of Follicle Stimulating Hormone (FSH), Luteinizing Hormone (LH), Activin-A, testosterone (T), Q10, estradiol (E2), and Antimullerian Hormone (AMH). The objective of the present study is to determine whether feeding CoQ10 in feed affects plasma hormone concentrations during non-breeding seasonality, which can be beneficial for improving semen quality in *Equus asinus*.

## 2 Materials and methods

This study was approved by the Research Committee of the Animal Policy and Welfare Committee of Liaocheng University (No. LC2019-1). The care and use of laboratory animals fully comply with local animal welfare laws, guidelines, and policies.

### 2.1 Experiment and animals

**Figure 1:**
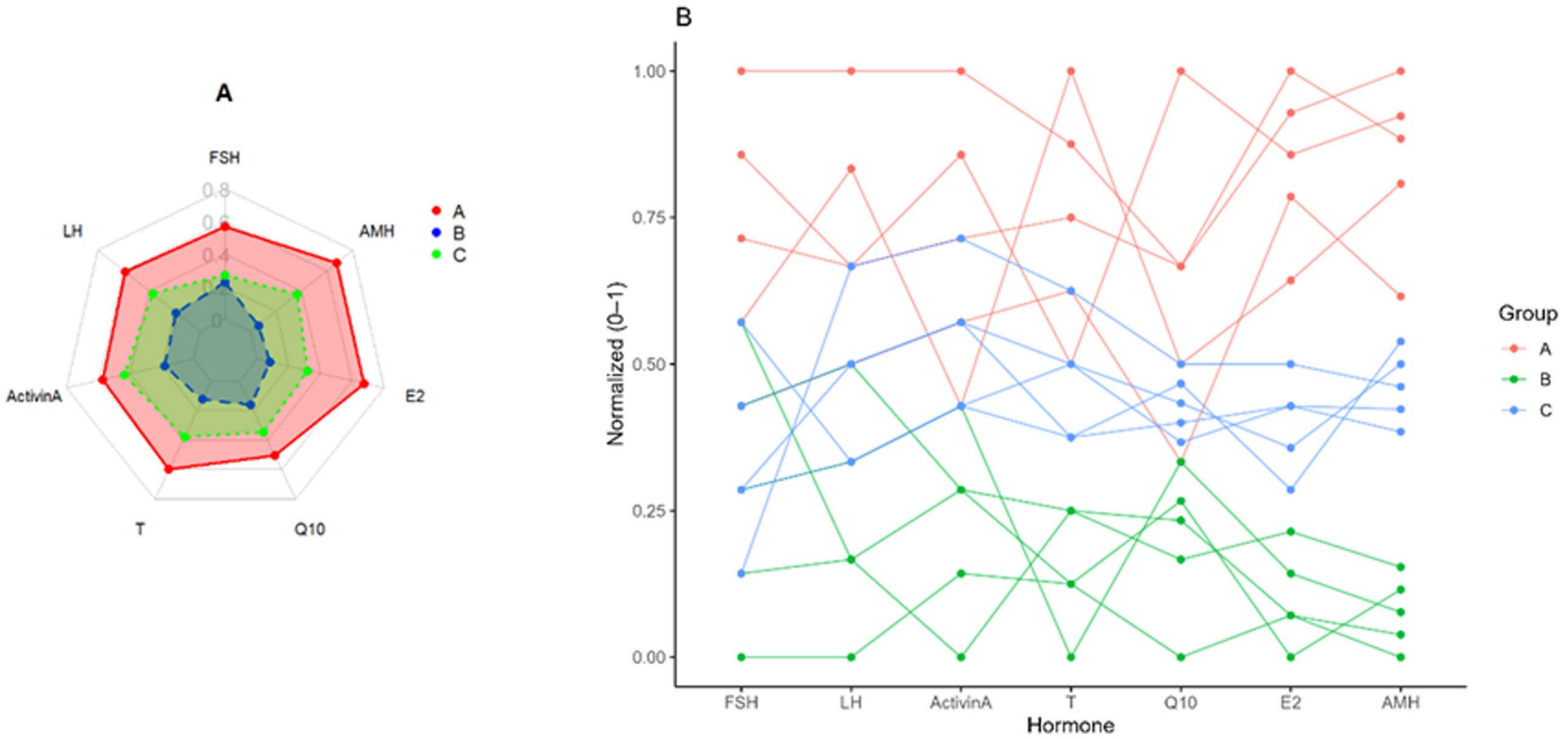
Endocrine profile patterns across the treated and control groups of FSH, LH, Activin-A, testosterone (T), Q10, estradiol (E2) and AMH. **(A)** Plot A: Radar plot (spider) of normalized mean hormone concentrations for each group. **(B)** Plot B: Parallel coordinate plot where each line represents an individual donkey across all measured hormones.

All jacks were offered silage and had free access to drinking water. The current experiment was conducted for 56 days, during non-seasonal breeding (November–January). The experiment was conducted at Liaocheng Wanshixing Breeding Co., Ltd. (E 115° and N 36°), Liaocheng, Shandong Province, China. Adult Dezhou jacks (*n* = 30), of the same genetic origin, with an average age of 2.5 ± 0.50 years, were randomly divided into three groups: A, B, and C. Each group had 10 animals (*n* = 10). Two grams of CoQ10-ubiquinol (Hebei Food Chemicals and Raw Materials Co, Ltd., China) were mixed into 1 pound of grain-based concentrate containing molasses. CoQ10 was given for 8 weeks. Current dose was used according to the manufacturer’s recommendations. All animals were kept under similar environmental conditions. For better administration of CoQ10, gelatin capsules (Hebei Food Chemicals and Raw Materials Co, Ltd., CHINA) were used. Each capsule contained 500 mg of CoQ10. Group A received 4 capsules, while Group B received 3. Jacks in group A were given 2 g/pound of grain-based diet/day. Animals in group B were given 1.5 g/pound of grain-based diet/day, and group C served as a control. All jacks (*n* = 30), in all groups, consumed grain-based concentrate having molasses, and there was no feed residual during the 56 days of the experimental trial. The experimental plan is shown in Figure 1.

### 2.2 Blood sampling

Blood samples were collected for plasma hormone analysis of T, Activin-A, LH, FSH, AMH, E2, and Q10. Blood samples were collected on the 21st, 28th, 34th, and 40th day of the experiment via the jugular vein into heparinized tubes. Within 3 hours of sample collection, plasma was separated from the blood by centrifugation at 1000 × *g* and kept at −20 °C until analysis.

### 2.3 Measurement of plasma hormone concentrations

Plasma Hormone and coenzyme Q10 concentrations were measured using commercial ELISA kits [MEIMIAN(r), Jiangsu Meimian Industrial Co., Ltd., Nanjing, China] and the protocols provided by the manufacturers. Measurement of testosterone (T) was performed using a bovine testosterone ELISA kit (Cat. No. MM-2382O1), with an assay sensitivity of 0.02 ng/mL and a range of 0.094–3.77 ng/mL. Inter and intra-assay coefficient of variation (CVs) were less than 10%. Activin-A was measured by ELISA (Cat. No. MM-51465O1), with a sensitivity of 0.4 ng/mL and CVs less than 10%. Assay Follicle-stimulating hormone (FSH): The ELISA kit was a horse-specific product (Cat. No. MM-34768O1) and had a sensitivity of 0.075 U/L and a detection range of 0.3–18 U/L. The intra- and inter-assay CVs were less than 10 and 10%, respectively. The assay used horse standard curves and reference standards provided. Luteinizing hormone (LH) was determined using a bovine LH ELISA kit (Cat. No. MM-34834O1); the sensitivity of the assay is 0.5 pg./mL, and the sensitivity is 2–50 pg./mL. Intra-assay CV was below 10%. ELISA (Cat. No. MM-51073O1) was used to measure anti-Mullerian hormone (AMH) concentration; the assay has a sensitivity of 0.05 ng/mL and a CV of less than 10%.

Estradiol (E2) concentration was measured using a bovine estradiol ELISA kit (Cat. No. MM-0023O1), with a sensitivity of 1 pmol/L and a detection range of 4–120 pmol/L; intra- and inter-assay CV were less than 10 percent. The measurement of Coenzyme Q10 (Q10) levels was performed with the help of a Coenzyme Q10 ELISA kit (Cat. No. MM-51501O1), and the sensitivity of the assay was 2.5 ng/mL, and the range of detection was 10–450 ng/mL. Interassay and intraassay CVs were less than 10%. All tests were conducted in accordance with the manufacturer’s instructions, ensuring uniformity and reliability of the measurements.

### 2.4 Statistical analysis

The Kolmogorov–Smirnov goodness-of-fit test was applied to determine normality. The data were transformed to logarithms when not normally distributed, and then re-tested for normality before analysis. Then, a two-way ANOVA was applied to compare mean values. A two-way repeated-measures ANOVA was performed to analyze the data, as consecutive samples from the same animal are not independent. The model evaluated the effects of treatment (A: 2 g CoQ10, B: 1.5 g CoQ10, C: control) and time on hormone levels, as well as the interaction between these factors. All the values were expressed as mean ± standard error of the mean (SEM). The differences across groups at various time points were analyzed using Bonferroni *post hoc* tests. Correlation analyses, PCA, and hierarchical clustering were conducted on the complete animal-level dataset to explore multivariate relationships and were not used for confirmatory hypothesis testing. The results were then visualized. To evaluate distribution patterns and treatment effects, raincloud plots were generated, incorporating half-violin density estimates, individual data points, and group mean ± SD boxplots for each hormone. By this approach, it was possible to visualize the distributional shape, variability, and central tendency simultaneously on the three groups. Radar charts are used to visually represent hormone response profiles on an integrative basis. As the seven hormones exist in various biological units and ranges, the values were first normalised across groups through min-max scaling (0 = the lowest observed group mean, 1 = the highest observed group mean per hormone). This process of normalisation ensured comparability across all axes and prevented the dominance of hormones with larger numerical scales. It follows that radar plots represent relative hormone balance across groups rather than absolute concentrations. All analyses were performed in R (version 4.4.3) using the *tidyverse*, *corrplot*, *ggplot2*, and *fmsb packages*. The probability levels *p* < 0.05 or 0.001 were set to determine significant differences among groups.

## 3 Results

Three groups, A, B, and C, were randomly assigned to experimental male donkeys. For 8 weeks, the animals in groups A and B received 2 g and 1.5 g of Ubiquinol, respectively, mixed into a molasses concentrate; however, the animals in group C served as a control and were fed only the concentrate. FSH, LH, Activin-A, testosterone (T), Q10, estradiol (E2), and AMH plasma concentrations were examined. To the best of our knowledge, this is the first study elaborating on various levels of CoQ10 on plasma hormone concentrations in donkeys during non-breeding seasonality.

**Figure 2:**
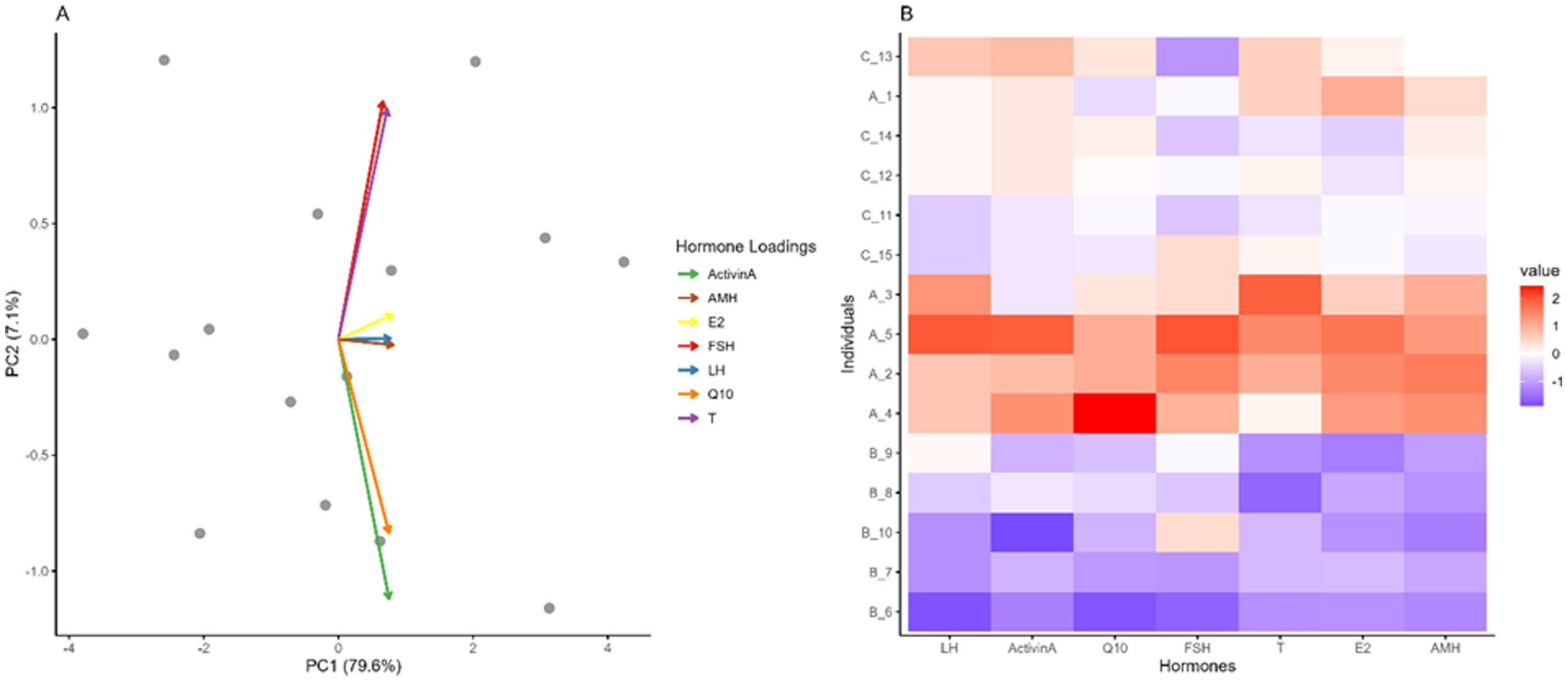
Exploratory multivariate analysis of hormone profiles across treated and control groups (FSH, LH, Activin-A, testosterone (T), Q10, estradiol (E2), and AMH). **(A)** Principal component analysis (PCA) biplot showing data distribution across the first two principal components: PC1 (79.6% variance explained) and PC2 (7.1% variance explained). Each point represents an individual observation. **(B)** Heatmap depicting hormone levels (LH, Activin-A, Q10, FSH, testosterone, E2, AMH) across individual samples labeled as A1, A2, B1, B2, etc.

The distribution density, variability, and hormone concentration data for groups A, B, and C are shown in panels A and B of Figure 2, with violin and box plots. Additionally, Panel A provides a clearer picture of individual data points, with error bars indicating heterogeneity within each group. The boxplot description, on the other hand, illustrates the distribution of data for each hormone among the several groups and represents the interquartile range (IQR). The median is shown by the line inside the box, which represents the interquartile range (IQR). Dots or other markers can be used to indicate outliers. The figure’s findings show that, for every hormone examined, there were notable variations in hormone concentrations among the three groups (A, B, and C). Panel B displays the distribution of these values using boxplots, while Panel A shows the concentrations of each hormone, with error bars indicating variability. Interestingly, concentrations of AMH and Activin A are higher than those of other hormones in all categories. While Group C has the lowest levels of most hormones, including FSH, LH, and Q10, Group A typically has higher concentrations of certain hormones, such as AMH and E2. The boxplots show how each group varies, with Group B showing a greater variation of hormone concentrations. These results imply that the groups had diverse hormonal profiles, which may reflect differences in physiological or experimental conditions.

**Figure 3:**
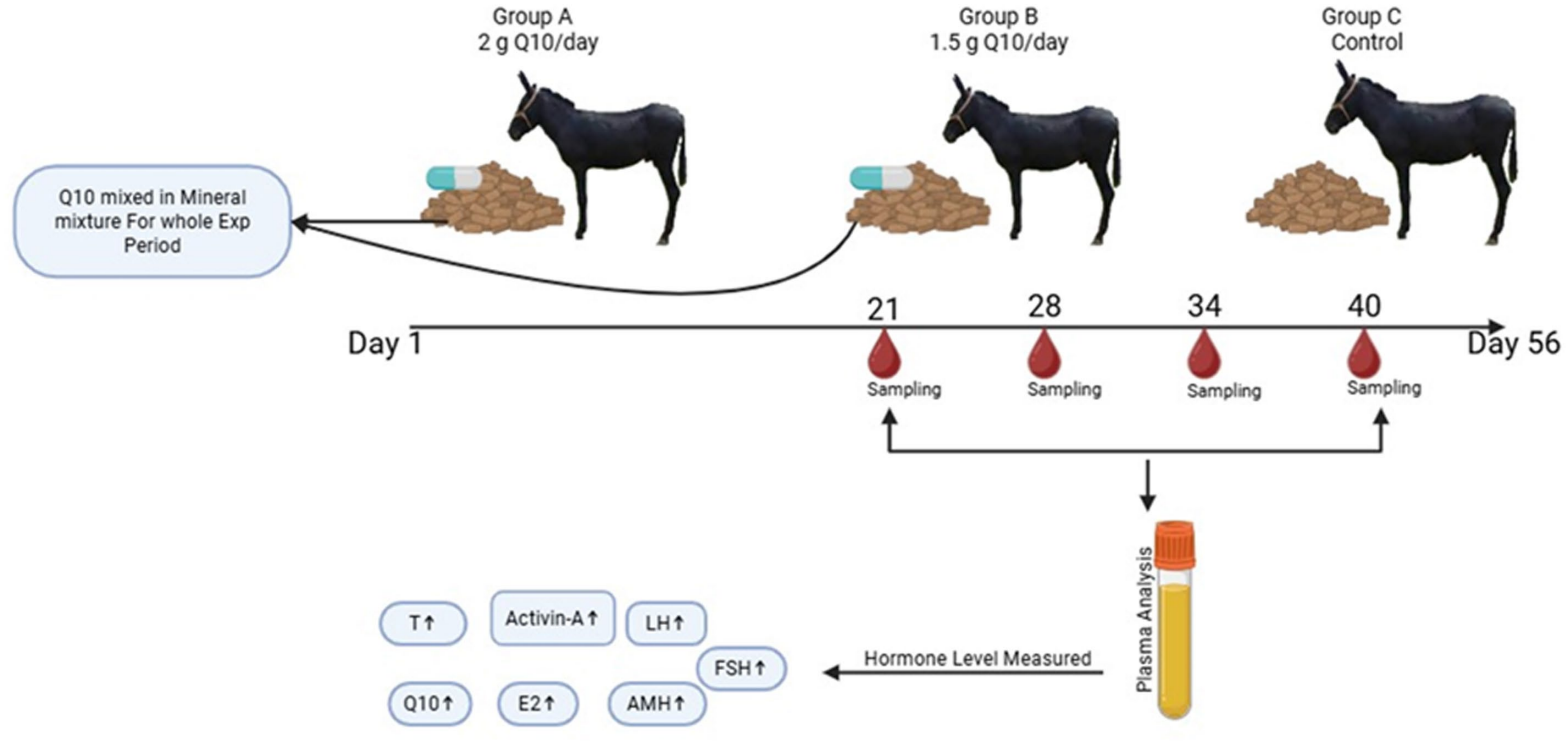
Grpahical illustration of three groups of Dezhou donkeys **Group A, B** and **C**. Group A was given Q10 2g/day, group B 1.5 g/day and group C was control. Experimental period lasted for 56 days. Blood sampling was performed on 21Ist, 28th, 34th and 40th days of experiment.

Figure 3 illustrates pairwise correlation coefficients between various variables, as displayed in the data in section A’s graph (heat map), where weaker correlations are represented by lighter hues and higher correlations (closer to 1.0) by darker reds. FSH, LH, Activin-A, testosterone (T), Q10, estradiol (E2), and AMH were the variables under comparison. Strong positive correlations between FSH, LH, Activin-A, testosterone (T), Q10, estradiol (E2), and AMH were found in part A, and they ranged from values of 0.8 and higher. Additionally, FSH and E2 show a moderate association (*r* = 0.78). Likewise, the connection between T and FSH (*r* = 0.54) is somewhat less than those of other couples.

**Figure 4:**
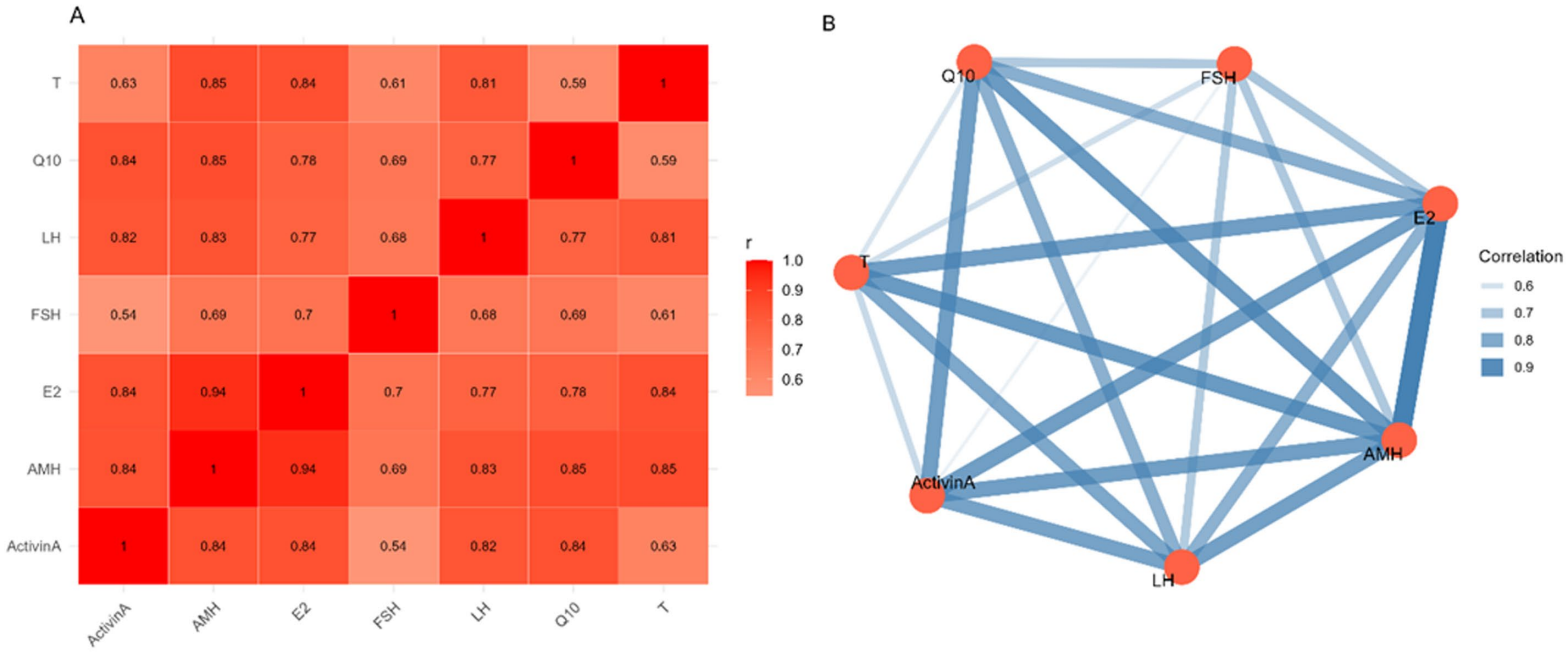
Inter-hormonal relationships in male donkeys across treatment groups for FSH, LH, Activin-A, testosterone (T), coenzyme Q10 (Q10), estradiol (E2), and AMH. **(A)** Pearson correlation heatmap showing pairwise associations among measured hormones. Color intensity represents correlation strength. **(B)** Hormone association network diagram illustrating correlations exceeding a threshold of *r* > 0.5. Nodes represent hormones, and edge thickness is proportional to the strength of the correlation.

The same correlation data is visualized as a radar plot and network in Part B (Network Plot) in Figure 4 (Supplementary material). The edges represent correlations exceeding a threshold (*r* > 0.5) among the variables T, Q10, LH, FSH, E2, AMH, and Activin A. A higher correlation is indicated by thicker edges (darker blue, which ranges from 0.6 to 1.0, implies stronger correlation). FSH and T, on the other hand, have a thinner edge, suggesting a lower association, while AMH and E2 have the thickest edge, suggesting a significant correlation.

Many of these factors show substantial positive associations, as demonstrated by the combined network plot and heatmap. Finding the factors most strongly correlated is easier with the network plot, which provides a clear depiction of these interactions. The correlation analysis results show strong positive correlations among most variables, suggesting they tend to rise or fall together. Interestingly, the strongest correlations are found between AMH and E2, followed by Q10, LH, and Activin A. These variables also show strong correlations with FSH and T. FSH and T have a comparatively lower association than in other couples, as shown by the heatmap and network diagram. Overall, the data reveal significant associations among these hormones and factors, particularly AMH and E2, which are strongly linked, suggesting possible underlying physiological or hormonal interactions.

Compared with groups B and C, group A’s results often show lower T and Q10 values and higher AMH and E2 values. With moderate levels for most markers, group B, on the other hand, shows a comparatively more balanced profile across the hormones. Furthermore, except for LH and T, which show values similar to those of group B, group C generally exhibits lower levels across all other markers, while showing exceptionally high values for Q10. This figure helps to compare the hormonal profiles of the three groups, indicating potential differences in biological or physiological responses related to these hormones. The radar plot (A) gives an overview of group profiles, while the line plot (B) provides a more detailed examination of how these levels change across hormones.

**Figure 5:**
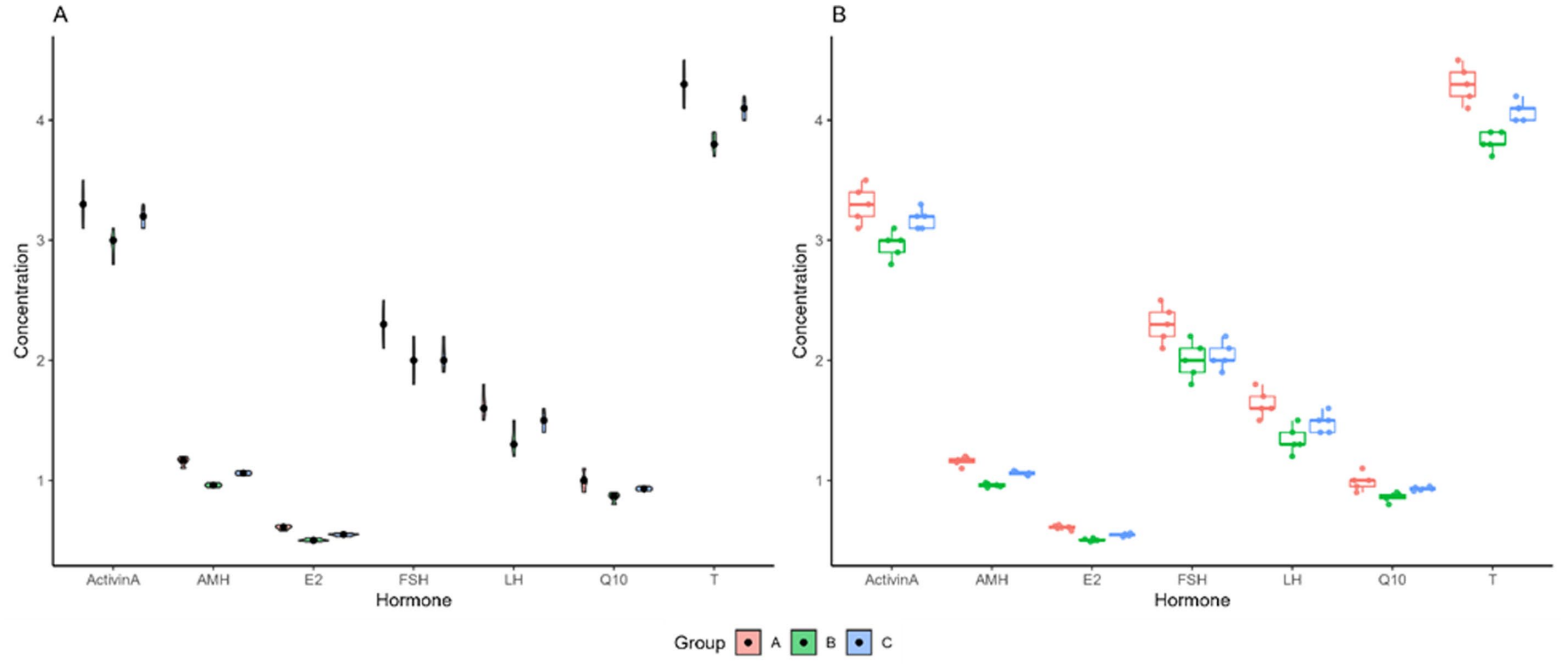
Distribution of plasma hormone concentrations in male donkeys across the groups of FSH, LH, Activin-A, testosterone (T), Q10, estradiol (E2), and AMH **(A)** Panel A: Violin plots showing distribution density and variability **(B)** Panel B: Boxplots with jittered points displaying individual variation in hormone concentrations.

Hormonal ratios among groups A, B, and C are shown by bar plots and scatter plots in Figures 5A,B (Supplementary material). The mean values (Activin/A AMH, LH/FSH, and T/E2) in this panel seem to be constant for groups A, B, and C, respectively. Group A has the highest mean levels of Activin/A AMH, while Groups B and C have lower levels that are still quite comparable. Additionally, there is a similar pattern in LH/FSH, with Group A exhibiting greater LH/FSH ratios than Groups B and C. All groups’ T/E2 values ranged widely, with Group A’s values being marginally higher than those of Groups B and C. In contrast, in a scatter plot, there appears to be a moderate positive correlation between T/E2 and LH/FSH, with Group A showing the highest spread and Group B showing intermediate values for all measures, and Group C consistently shows the lowest values for Activin/AAMH and LH/FSH, though T/E2 shows a scattered trend.

The PCA plot’s point distribution indicates that PC1, which is likely driven by differences in hormone levels such as Activin A and AMH, is the most important factor separating the individuals. The spots’ relative locations along PC2 might reveal more minute variations in hormone profiles. Each person’s unique hormonal profile is displayed in the heatmap. The grouping of people with comparable hormone levels can reveal patterns or groupings in the data that may be associated with physiological or illness states. The red-shaded cells indicate that A_4 has higher FSH and LH levels than the others. In a similar vein, A_9 appears to have higher AMH levels than others, suggesting a potential unique hormonal profile. Finding biomarkers or predictions for certain illnesses can also be aided by analyzing individual differences in hormones. The findings show that there is individual hormonal variability, and these differences play a major role in the patterns seen in the heatmap and PCA plot. Positive associations among hormonal variables were observed across the dataset, with correlation coefficients ranging from moderate to high. These patterns are presented for exploratory and descriptive purposes.

## 4 Discussion

Coenzyme Q10 (CoQ10) in its reduced form, Ubiquinol, is a strong lipophilic antioxidant that can regenerate other antioxidants, including vitamins E and C (25). Peroxyl radicals produced during lipid peroxidation are also efficiently scavenged (26). The formulation and dosage utilized have a significant impact on the bioavailability of both Ubiquinol and ubiquinone (27). Moreover, prior to absorption, ubiquinone must be enzymatically reduced to Ubiquinol, as the majority of oral CoQ10 is absorbed in its reduced form (28). Therefore, after Ubiquinol supplementation, plasma CoQ10 concentrations are higher because powdered Ubiquinol has better intestinal absorption efficiency than ubiquinone (25).

Heat stress is being used as a model for oxidative damage. Male fertility and animal productivity are significantly reduced by environmental heat stress (29). Several studies have shown that antioxidant supplements can reduce heat stress-induced male infertility because oxidative stress is the main mechanism causing heat-induced testicular dysfunction (29–31). In addition, CoQ10 and other mitochondria-targeted antioxidants may provide better protection than cytosolic antioxidants, since mitochondria are the main sites of reactive oxygen species (ROS) generation (32). In seminiferous tubule morphology, such as diameter, epithelial height, and thickness, CoQ10 supplementation has been demonstrated to preserve these parameters (33). Furthermore, CoQ10 administration significantly migitates the negative effects of heat stress on testicular architecture and spermatogenic cells in rats, restoring testicular weight due to its powerful anti-apoptotic and antioxidant characteristics (34).

The impact of daily Ubiquinol supplementation on male donkey plasma CoQ10 concentrations has not yet been reported. Furthermore, after giving horses 800 mg of ubiquinone orally for 60 days, Hosoe et al. (35) found that plasma CoQ10 levels were much higher than in humans who received 90 mg of emulsified Ubiquinol daily for 4 weeks, mainly due to metabolic differences (36). For 8 weeks, male donkeys in the current study were randomly assigned to groups A and B, which received 2 g and 1.5 g of Ubiquinol, respectively, combined with concentrate and molasses. Group C served as the control.

The results showed significant intergroup variation in circulating reproductive hormone levels, with Activin A and AMH showing the highest levels overall. Notably, donkeys in group A that received 2 g of Ubiquinol supplement had higher levels of estradiol (E2) and AMH. The current study, which is consistent with earlier research, demonstrated that oral CoQ10 supplementation improves testicular hemodynamics, semen characteristics, testosterone production, and seminal antioxidant capacity in goats while mitigating the effects of summer heat stress (37). In contrast, supplementation with CoQ10 has been shown to enhance Sertoli cell function by promoting the release of AMH and E2. Furthermore, the FSH-dependent aromatization of testosterone in Sertoli cells produces estradiol-17β (38). However, no appreciable changes in FSH, LH, or E2 levels were noted after supplementation of CoQ10, which is probably because gonadotropin secretion is pulsatile (39). On the other hand, some data suggest that CoQ10 supplementation may raise prolactin levels while decreasing FSH and LH levels, which may be a reflection of hyperprolactinemia’s anti-gonadotropic effects (40, 41). Further investigation is required to determine if these mechanisms contribute to the hormonal profiles observed during the non breeding season.

Additionally, the current investigation showed that the FSH, LH, and CoQ10 concentrations were lowest in the control group (C). One could argue that, compared with the untreated group, oral coenzyme Q10 supplementation has helped protect the testicles of male donkeys. Testicular and hypothalamic–pituitary-gonadal (HPG) axis activity can be better understood by tracking circulating reproductive hormones (42, 43). In contrast to groups B and C, group A showed lower levels of CoQ10 and testosterone (T). This could be the effects of CoQ10 that might not directly control the production of T or CoQ10 (44). Furthermore, as CoQ10 is essential for electron transport and mitochondrial bioenergetics, which support steroidogenic activity, the greater T concentrations shown in group B (1.5 g CoQ10) might reflect improved Leydig cell function (34). Group B in the current study showed significant, balanced alterations in their hormonal profiles, with moderate levels of Q10, LH, and T. This indicates that the HPG axis is optimally coordinated. By improving mitochondrial and antioxidant activity, these moderately higher plasma CoQ10 concentrations may promote spermatogenesis and steroidogenesis. Additionally, CoQ10 and L-carnitine work together to improve spermatogenesis, antioxidant activity, and sex hormone secretion (40). Another study demonstrated that improved LH and T levels are associated with better semen quality (45). In the hypothalamic–pituitary-testicular axis, we know that LH directly affects T, and our experiment yielded similar results. Along with plasma hormone concentrations of LH and T, Q10 was also elevated. It clearly states that improved plasma LH, T, and Q10 may ensure better semen quality in male donkeys, thereby ultimately improving reproductive efficiency. Thus, 1.5 d/day CoQ10 has greater beneficial effects, as shown in group B, compared with 2 g/day in group A.

Furthermore, it has been shown that lipid-soluble vitamins, such as CoQ10, are essential for both antioxidant defense and mitochondrial energy production (46, 47). Because it is hydrophobic, its bioavailability is low even after endogenous synthesis, whereas Ubiquinol, the reduced form, has stronger biological antioxidant activity (48, 49). Furthermore, infertile men have a significantly lower seminal CoQ10 concentration, which decreases fertility; dietary supplementation successfully raises seminal CoQ10 levels and enhances sperm motility and density (40, 50). Furthermore, there is a favorable correlation between sperm concentration and motility and the levels of CoQ10 in seminal plasma (51, 52). Hormonal ratio analysis in this study showed that group A had higher mean values for T/E2, LH/FSH, and activin-A/AMH, with the T/E2 and LH/FSH ratios showing particularly pronounced fluctuations. The Activin-A/AMH ratio reflects the balance between follicular stimulation (Activin-A) and follicle pool regulation (AMH); if first reported in donkeys, it represents a novel endocrine indicator of ovarian activity. An increase in AMH during the non-breeding season suggests the persistence of a substantial pool of small follicles despite reduced reproductive drive.

The concurrent rise in estradiol indicates that follicles remain steroidogenically active outside the breeding season, highlighting distinctive seasonal reproductive physiology in donkeys. According to a recent study, Pb-free rats supplemented with CoQ10 had significantly higher levels of testosterone and LH, but unchanged FSH levels, compared with the control group. It is interesting to note that sex hormone levels significantly increased in groups treated with PbAc and CoQ10 (53). However, previous research also suggested that feedback modulation of the hypothalamic–pituitary-gonadal axis may be the cause of decreases in gonadotropins (FSH and LH) after taking CoQ10 supplements (33, 51). Above all these facts, we must consider that seasonal breeding is a natural phenomenon, and it’s not so simple to modulate major players of the HPG axis in seasonal breeding.

In addition, CoQ10 is mostly eliminated through urine and feces (27), and it is mostly produced in metabolically active tissues such as the liver, heart, kidneys, and skeletal muscles (54). The heatmap (Part A) shows that most discoveries, including Q10 and reproductive hormones, exhibit strong positive associations (*r* ≥ 0.8). These results are consistent with earlier research showing that CoQ10 supplementation in males with idiopathic oligoasthenoteratozoospermia improves sperm concentration, motility, and antioxidant capacity (55). Additionally, oxidative stress is a component in male infertility, and optimal sperm activity depends on balanced reactive oxygen species (ROS) levels (56). In contrast, excessive ROS production disrupts the antioxidant balance, damages sperm structure via oxidative damage, and eventually reduces fertility (55, 57). In addition, CoQ10 supplementation mitigates the testicular dysfunction associated with chronic kidney disease by enhancing sperm quality, testicular architecture, testosterone synthesis, and antioxidant defense mechanisms (58).

In the network plot (Part B), it is noteworthy that AMH and E2 have the strongest connection, but FSH and T have a weaker linkage. Overall, both approaches show that hormone interactions are closely linked, indicating coordinated physiological control. Moreover, CoQ10 and LS supplements have been shown to have synergistic effects on semen quality, improving sperm function and sexual behavior. Furthermore, these gains are partially ascribed to the hypothalamic–pituitary-gonadal (HPG) axis being modulated by increased secretion of LH, FSH, and testosterone, as well as by upregulated GnRH expression (40). Infertility or sexual desire disorder can also arise from any disturbance in these axes (59). All facets of sexual behavior are positively impacted when LS and CoQ10 are administered together (40). In addition, oral CoQ10 supplementation can boost fertility factors such as sperm count and motility, and elevate the sexual hormone levels and spermatogenesis in rats (50). In this study, elevated activin A levels observed in PCA and heatmap analyses suggest that CoQ10 supplementation modulates testicular function and secretory activity, potentially enhancing seminal quality in male donkeys. Furthermore, activin releases stimulate FSH secretion from pituitary gonadotropes (60) and contribute to testicular development and adult reproductive capacity, a process modulated by antagonists such as FSTL3 (61). According to this study, increased activin A levels found in PCA and heatmap analyses imply that CoQ10 supplementation alters secretory activity and testicular function, which may improve the quality of male donkey seminal tissue. Additionally, activin releases promote pituitary gonadotropes to secrete FSH (60) and aid in testicular growth and adult reproductive potential, a process that is influenced by antagonists such as FSTL3 (61).

Individual physiological variability was evident in the different hormonal profiles observed in this investigation, with higher AMH in group A_9 and elevated FSH and LH in group A_4. These outcomes are consistent with earlier research demonstrating that supplementing both CoQ10 and LS leads to GnRH overexpression, which, in turn, increases FSH and LH secretion. The current hormonal clustering pattern findings suggest potential biomarkers of reproductive regulation. Improvements in sperm motility, viability, and sexual activity may also be attributed to CoQ10 + LS supplementation’s apparent modulation of the HPG axis, leading to increased GnRH and LH production (40). Thus, CoQ10 has a beneficial effect on the metabolism and production of reproductive hormones (62). Reduced CoQ10 activity or levels impair cellular antioxidant capability, resulting in the accumulation of free radicals that negatively impact fertility and sexual behavior (63). Furthermore, mitochondria-rich organs such as the heart, lungs, and adrenal glands contain high concentrations of the androgenic, lipid-soluble compound CoQ10 (64). The findings of the present study further support the notion that CoQ10 enhances testicular antioxidant activity and improves male reproductive function (64, 65, 66). Molecular regulation of CoQ10 needs to be explored further for in-depth outcomes.

## 5 Conclusion

Oral supplementation of Ubiquinol showed promising results in improving plasma hormone concentrations in donkeys. Two gram/day of Ubiquinol mixed in grain-based concentrate with molasses improved plasma hormones related to better reproductive performance, and it may also aid to improve semen quality. Further studies must focus on analyzing semen quality and exploring molecular mechanisms, including Sertoli and Leydig gene expression levels.

## Data Availability

The original contributions presented in the study are included in the article/Supplementary material, further inquiries can be directed to the corresponding authors.

## Supplementary material

Supplementary Figure S1 Hormonal ratios and derived indices across the treated and control groups of FSH, LH, Activin-A, testosterone (T4), Q10, estradiol (E2) and AMH. **(A)** Plot A: Bar plots of T/E2, LH/FSH, and Activin-A/AMH ratios (mean ± SEM) **(B)** Plot B: Scatterplot of T/E2 versus LH/FSH ratios to explore clustering of individuals.
