# Supplementary figures and images for: Effects of varying concentrations of Ubiquinol (coenzyme Q10) on physiological parameters in male donkeys (Equus asinus)
Source: Front Vet Sci. 2026 Feb 16;13:1733317. doi: 10.3389/fvets.2026.1733317 (PMC12951480; doi:10.3389/fvets.2026.1733317)

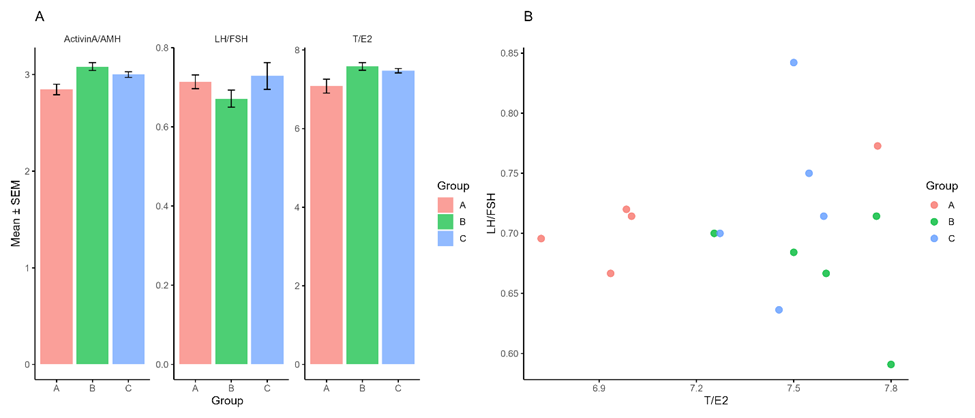

Supplement: Supplementary Figure S1 — Hormonal ratios and derived indices across the treated and control groups of FSH, LH, Activin-A, testosterone (T4), Q10, estradiol (E2) and AMH. (A) Plot A: Bar plots of T/E2, LH/FSH, and Activin-A/AMH ratios (mean ± SEM) (B) Plot B: Scatterplot of T/E2 versus LH/FSH ratios to explore clustering of individuals. [file Image_1.tiff]
